# RNA-seq Analysis of Overexpressing Ovine *AANAT* Gene of Melatonin Biosynthesis in Switchgrass

**DOI:** 10.3389/fpls.2016.01289

**Published:** 2016-08-31

**Authors:** Shan Yuan, Yanhua Huang, Sijia Liu, Cong Guan, Xin Cui, Danyang Tian, Yunwei Zhang, Fuyu Yang

**Affiliations:** ^1^College of Animal Science and Technology, China Agricultural UniversityBeijing, China; ^2^College of Agriculture, China Agricultural UniversityBeijing, China; ^3^Beijing Key Laboratory for Grassland Science, China Agricultural UniversityBeijing, China; ^4^National Energy R&D Center for BiomassBeijing, China; ^5^Beijing Sure Academy of BiosciencesBeijing, China

**Keywords:** switchgrass, melatonin, overexpression, o*AANAT*, RNA-seq

## Abstract

Melatonin serves important functions in the promotion of growth and anti-stress regulation by efficient radical scavenging and regulation of antioxidant enzyme activity in various plants. To investigate its regulatory roles and metabolism pathways, the transcriptomic profile of overexpressing the ovine arylalkylamine *N*-acetyltransferase (*oAANAT*) gene, encoding the penultimate enzyme in melatonin biosynthesis, was compared with empty vector control using RNA-seq in switchgrass, a model plant of cellulosic ethanol conversion. The 85.22 million high quality reads that were assembled into 135,684 unigenes were generated by Illumina sequencing for transgenic o*AANAT* switchgrass with an average sequence length of 716 bp. A total of 946 differentially expression genes in transgenic line comparing to control switchgrass, including 737 up-regulated and 209 down-regulated genes, were mainly enriched with two main functional patterns of melatonin identifying by gene ontology analysis: the growth regulator and stress tolerance. Furthermore, KEGG maps indicated that the biosynthetic pathways of secondary metabolite (phenylpropanoids, flavonoids, steroids, stilbenoid, diarylheptanoid, and gingerol) and signaling pathways (MAPK signaling pathway, estrogen signaling pathway) were involved in melatonin metabolism. This study substantially expands the transcriptome information for switchgrass and provides valuable clues for identifying candidate genes involved in melatonin biosynthesis and elucidating the mechanism of melatonin metabolism.

## Introduction

Since melatonin (*N*-acetyl-5-methoxytryptamine) was first discovered in plant in Dubbels et al. (1995) and Hattori et al. (1995), varying concentrations of melatonin have been found in many other plant species (Murch et al., 1997; Manchester et al., 2000; Simopoulos et al., 2005). The fundamental issues of melatonin biosynthetic pathways and physiological functions still need to be deciphered to utilize in plants (Tan et al., 2003, 2012; Arnao and Hernández-Ruiz, 2009; Arnao, 2014; Wei et al., 2016). Both exogenous melatonin treatments (Kolár et al., 2003; Arnao and Hernández-Ruiz, 2007; Sarropoulou et al., 2012; Zhang et al., 2013) and melatonin-rich transgenic plants (Kang et al., 2010; Park et al., 2012; Byeon and Back, 2014; Wang et al., 2014) have been carried on in order to determine its potential functional roles. The physiological role of melatonin in plants involved growth regulation, scavenging reactive oxygen species and increases of antioxidant enzyme activities (Paredes et al., 2008; Arnao and Hernández-Ruiz, 2014; Hardeland, 2016). Through the encoding genes for the catalytic reactions during melatonin biosynthesis have been identified and cloned recently, its biosynthetic pathways still not clearly deciphered yet in plants (Kang et al., 2011, 2013; Byeon et al., 2013a; Hardeland, 2016). The classic pathways of melatonin biosynthesis consist of the four catalytic reactions from tryptophan: decarboxylation by tryptophan decarboxylase (TDC), hydroxylation by tryptamine 5-hydroxylase (T5H) to serotonin, *N*-acetylation by serotonin *N*-acetyltransferase (SNAT) and the final *O*-methylation to melatonin by *N*-acetylserotonin *O*-methyltransferase (ASMT). The penultimate step catalyzes the same reaction as the non-homologous AANAT of vertebrate, which catalyzes conversion of serotonin into *N*-acetylserotonin (Byeon et al., 2013a). Overexpression of *AANAT* gene can promote the ability of biosynthesis of melatonin, and significantly improve the melatonin content in plants (Kang et al., 2010; Wang et al., 2014; Zhang et al., 2014).

Switchgrass (*Panicum virgatum* L.) is a perennial C4 grass native to North America, and is well-researched on germplasm collection, cultivation, genetic breeding as a model plant for cellulosic bioethanol production over the past several decades (Sanderson et al., 1996; Parrish and Fike, 2005). Aiming at the production demand of large biomass, the traditional breeding techniques have been challenged by its nature of outcrossing and polyploidy, as well as the general infertile environments for the biomass grass cultivation. Fortunately, modification of the functional genes that related to the growth and resistance could effectively assist the breeding process of switchgrass (Fu et al., 2011; Poovaiah et al., 2014; Baxter et al., 2015; Wuddineh et al., 2015). Several transgenic plants expressing the key genes for melatonin biosynthesis enzymes have been demonstrated to increase the resistance to environmental stresses (Kang et al., 2010; Park et al., 2012).

In this study, the transgenic melatonin-rich switchgrass (overexpressing of the *oAANAT* gene) and the transgenic control with empty vector (EV) were used to conduct a RNA-seq and analyze the effects of melatonin on gene expression. This research intends to provide differentially expressed genes (DEGs) in the melatonin-rich switchgrass and mechanism information on the biosynthesis, regulation, metabolism of melatonin for further investigations in plants.

## Materials and Methods

### Plant Materials and Cultivation

The switchgrass (*Panicum virgatum* L. var. Alamo) plants expressing sheep *AANAT* gene (ID: 25120) were used for this study. The transgenic plants were grown under 16 h light (26°C, 120 μmol/m^2^/s) and 8 h dark (18°C) conditions with watered every other day. Fully matured plants were chosen from each genotype for molecular characterization and transcriptome sequencing, which were all phenotypically identical with respect to overall size, tiller number and leaf pattern. Three replicates of relative high melatonin contents for each transgenic stem were frozen in liquid nitrogen and stored at 80°C until further analysis.

### Characterization of Growth and Development of Transgenic Switchgrass

**FIGURE 1:**
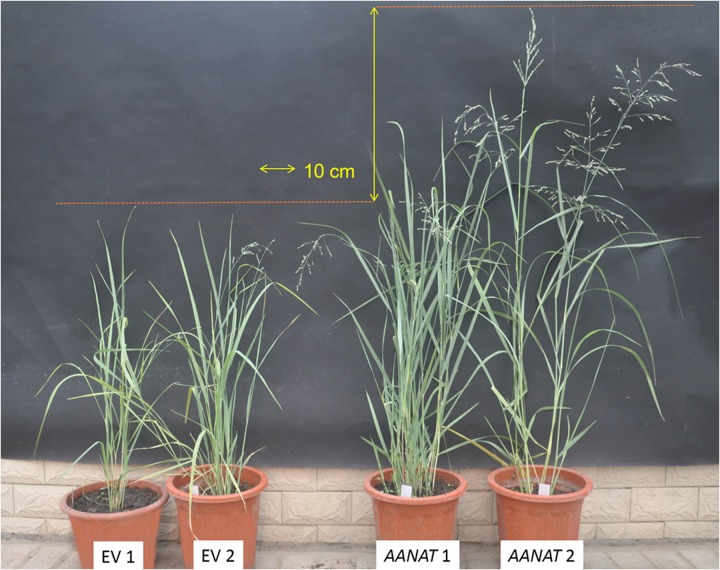
**The phenotypes of transgenic *oAANAT* line and the control switchgrass in reproductive third stage**.

Tiller number, plant height, internode number, internode length, internode diameter, leaf blade length, leaf blade width, root number, root length, root diameter, and spike length were measured at the transgenic reproductive third (R3) stage (**Figure 1**), a stage with fully emerged spikelets and an emerged peduncle (Hardin et al., 2013). Internode 3 (I3) was used for measuring internode diameter. The leaves of I3 were used to measure leaf blade length and leaf blade width. Twelve replicates were measured for each transgenic line.

### RNA Isolation and Qualification

Total RNA was extracted from the stems using the TRIzol reagent method (Invitrogen, Carlsbad, CA, USA) and was treated with RNase-free DNase or 30 min at 37°C to remove residual DNA. RNA degradation and contamination was monitored on 1% agarose gels. RNA purity was checked using the NanoPhotometer spectrophotometer (Implen, Westlake Village, CA, USA). RNA concentration was determined by Qubit RNA Assay Kit in Qubit 2.0 Flurometer. RNA integrity was assessed with the RNA Nano 6000 Assay Kit of the Agilent Bioanalyzer 2100 system (Agilent Technologies, Santa Clara, CA, USA).

### Transcriptome Sample Preparation for Sequencing

The total amount of 1.5 μg RNA per sample was prepared for the RNA-seq. Sequencing libraries were generated using NEBNext Ultra^TM^ RNA Library Prep Kit for Illumina (NEB, USA). The cDNA fragments of 150∼200 bp were preferentially selected from the library by purification with AMPure XP system (Beckman Coulter, Beverly, MA, USA).

### Clustering and Sequencing

The clustering of the index-coded samples was performed on a cBot Cluster Generation System using TruSeq PE Cluster Kit v3-cBot-HS (Illumia). After cluster generation, paired-end reads were generated by sequencing with the library preparations on an Illumina Hiseq platform.

### Validation of RNA-seq Data by Real-Time Quantitative

Real-time quantitative PCR validation of RNA-seq data for 12 random genes was performed in 20 μl of reaction mixture (**Supplementary Table S1**). The amount of the amplified DNA was monitored by fluorescence at the end of each cycle using 7500 Real-Time PCR System (Applied Biosystems). Each plate was repeated three times in independent runs for all reference and selected genes. Gene expression was evaluated by the 2^-ΔΔCt^ method (Livak and Schmittgen, 2001).

### Data Analysis

#### Quality Control

Clean data (clean reads) were obtained by removing reads containing adapter, reads containing ploy-N and low quality reads from raw data. Q20, Q30, GC-contents and sequence duplication level of the clean data were calculated. Our raw data have been uploaded to NCBI^1^, and the accession number is PRJNA322585.

#### Transcriptome Assembly

The complete genome of switchgrass has not been released through the draft version was released in 2012 and updated to V3. *De novo* transcriptome assembly was accomplished using Trinity (Grabherr et al., 2011) with min_kmer_cov set to 2 by default and all other parameters set default.

#### Gene Functional Annotation

Gene function was annotated based on the following databases: Nr (NCBI non-redundant protein sequences); Nt (NCBI non-redundant nucleotide sequences); Pfam (Protein family); KOG/COG (Clusters of Orthologous Groups of proteins); Swiss-Prot (A manually annotated and reviewed protein sequence database); KO (KEGG Ortholog database); GO (Gene Ontology).

#### Differential Expression Analysis

Gene expression levels were estimated by RSEM (Li and Dewey, 2011). Differential expression analysis of two conditions/groups was performed using the DESeq R package (1.10.1). Genes with an adjusted *P*-value < 0.05 found by DESeq were assigned as differentially expressed using the Benjamini and Hochberg’s approach.

#### GO Enrichment Analysis and KEGG Pathway Enrichment Analysis

GOseq R packages were implemented to define the DEGs based Wallenius non-central hyper-geometric distribution (Young et al., 2010). We used KOBAS (Mao et al., 2005) software to test the statistical enrichment of differential expression genes in KEGG pathways^2^ (Kanehisa et al., 2008).

## Results

### Morphological Characterization of Transgenic Plants

The transgenic switchgrass exhibited the largely enhanced growth condition (shoot, leaf, and root) compared with the EV (**Figure 1**; **Table 1**). Average tillers, shoot height, stem nodes, third internode length, and stem diameter in transgenic line were 45.5, 80.2, 48.3, 63.3, and 22.99% higher than those of EV, respectively (*P* < 0.05). Leaf blade length was 23.4% longer in transgenic switchgrass (52.72 cm) than that of EV (43.53 cm, *P* < 0.05). There was no significant difference on leaf width between the two groups (*P* > 0.05). Average roots, root length, and root diameter were 24.2, 25.6, and 41.5% higher in melatonin-rich switchgrass than those of control groups. Mean spike length in *AANAT* transgenic switchgrass (23.17 cm) was more than fourfold of the control (5.4 cm, *P* < 0.05; **Table 1**).

**Table 1:** Morphological characterization of transgenic switchgrass plants in reproductive third stage.

	Tiller number	Plant height (cm)	Stem node number	Internode length (3) (cm)	Internode diameter (cm)	Leaf blade length (cm)	Leaf blade width (cm)	Root number	Root length (cm)	Root diameter (cm)	Spike length (cm)
EV	6.1 ± 0.4b	66.81 ± 4.15b	3.2 ± 0.44b	8.78 ± 0.61b	2.95 ± 0.23b	43.53 ± 2.76b	1.17 ± 0.10a	16.5 ± 1.35a	41.95 ± 0.35b	0.99 ± 0.06b	5.40 ± 2.31b
*A*	8.9 ± 0.4a	120.37 ± 8.94a	4.8 ± 0.38a	14.33 ± 0.81a	3.63 ± 0.30a	53.72 ± 0.51a	1.13 ± 0.11a	20.5 ± 1.71a	52.68 ± 3.51a	1.4 ± 0.10a	23.17 ± 2.86a


*EV: expressing the empty vector only, *A*: transgenic *oAANAT* line. Different letters indicate significant differences (*P* < 0.05).*

### Illumina Paired-End Sequencing and Assembly

Total RNA was extracted from transgenic and control switchgrass in order to sequence using Illumina paired-end sequencing technology. In this study, exceeded 74G clean bases were acquired, and the average GC-rich content and the Q30 level of the six samples was 58.45 and 93.64%, respectively (**Supplementary Table S2**). After removal of adaptors and low-quality reads, average of 85,227,460 and 81,104,143 clean reads (95.9 and 94.2% of the raw data) of transgenic line and control were obtained, respectively (**Supplementary Table S2**). These reads were assembled into 264,869 transcripts with an average length of 1,052 bp and an N50 of 1,792 bp. After compared the different transcripts representing one unigene, the longest length transcript for each unigene was extracted. A total of 135,684 unigenes were obtained. The average length was 716 bp, and transcripts with lengths of more than 500 bp accounted for about 37.65% of all transcripts (**Supplementary Table S3**).

### Annotation of All Non-redundant Unigenes

For the validation and annotation of the assembled unigenes, all assembled unigenes were submitted to a BLASTx search with an E value threshold of 1e-5 against the following databases: Nr (NCBI non-redundant protein sequences), Nt (NCBI non-redundant nucleotide sequences), Pfam (Protein family), Swiss-Prot (a manually annotated and reviewed protein sequence database), GO (Gene Ontology), KOG (eukaryotic orthologous groups) and KEGG (Kyoto Encyclopedia of Genes and Genomes). The unigenes were subjected to public databases for similarity searching. Among 135,684 unigenes, 56,968 (41.98%), 605,126 (44.59%) and 38,266 (28.2%) unigenes showed homology with sequences in the NCBI Nr, Nt and SwissProt databases, respectively (**Supplementary Table S4**; **Supplementary Figure S1**).

### Functional Classification by GO and KOG

Gene ontology (GO, a standardized classification system for gene function) was assigned to classify the functions of predicted switchgrass unigenes. In total, 1,931 functional GO terms were assigned among 41,494 unigenes with BLAST matching to known proteins (**Supplementary Table S4**). The majority of the unigenes were assigned to the categories of biological processes (100,887, 47.61%), followed by cellular components (63,207, 29.83%) and molecular functions (47,821, 22.57%; **Supplementary Figure S2**). Under the category of biological processes, cellular processes (22,564, 22.37%) and metabolic processes (21,358, 21.17%) were prominently represented. Under the classification of molecular functions, the binding (22,620, 47.3%) and catalytic activities (17,760, 37.1%) represented the two largest categories, while other categories, such as those for transporter activities, structural molecule activity, molecular function regulator, and others, together contained only 7,441 unigenes representing 15.56% of the total number of unigenes. As for the cellular component, two categories, pertaining to cells and cell parts, accounted for approximately 40.38% of the cellular components that were identified; the organelle category accounted for approximately 13.53% of the cellular component unigenes, and the membrane and membrane part categories accounted for 19.43%.

In order to predict and classify possible functions, all unigenes were aligned to the euKaryotic Ortholog Groups (KOG) database in which orthologous gene products were classified. Out of 56,968 unigenes with significant similarity to nr proteins in this study, 21,165 sequences were assigned to KOG classifications. Among the 26 KOG categories, the cluster related to general function prediction (3,674, 17.36%) was the largest group, followed by those for post-translational modification (2,670, 12.62%); translation, translation, ribosomal structure, and biogenesis (1,693, 8.00%); and signal transduction mechanisms (1,605, 7.58%).

### Functional Classifications Using KEGG Pathways

**FIGURE 2:**
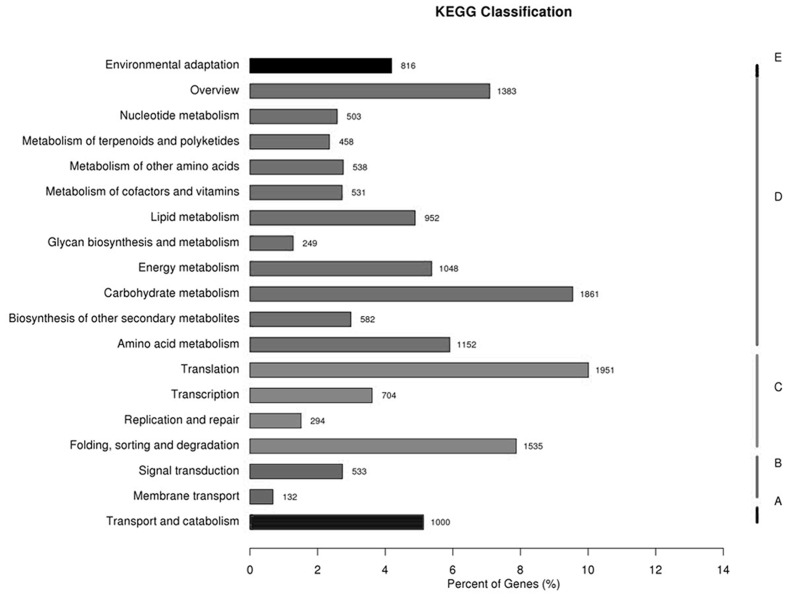
**Pathway assignment based on the Kyoto Encyclopedia of Genes and Genomes (KEGG) database.** (A) Classification based on cellular process categories, (B) classification based on environmental information processing categories, (C) classification based on genetic information processing categories, (D) classification based on metabolism categories, and (E) classification based on organismal systems categories.

All the unigenes were analyzed with respect to the KEGG pathway database to further examine the transcriptome of transgenic switchgrass. Out of the 135,684 identified unigenes, 16,222 (11.96%) with significant matches were assigned to five main categories that included 131 KEGG pathways (**Figure 2**; Supplementary file-KEGG). Among the five main categories that were identified, metabolism held the greatest number of unigenes (9,257, 57.06%), followed by genetic information (4,484, 27.64%), cellular processes (1,000, 6.17%), organismal systems (816, 5.03%), and environmental information processing (665, 4.10%). These results indicate that active metabolic processes were occurring in transgenic switchgrass. As shown in Supplementary file-KEGG, the metabolism category contained 19 sub-categories, including environmental adaptation, nucleotide metabolism, metabolism of terpenoids and polyketides, energy metabolism, carbohydrate metabolism, folding, sorting and degradation, membrane transport, biosynthesis involved in other secondary metabolism, amino acid metabolism, transport and catabolism, signal transduction and (**Figure 2**).

### Differential Gene Expression in Melatonin-Rich Transgenic Switchgrass

**FIGURE 3:**
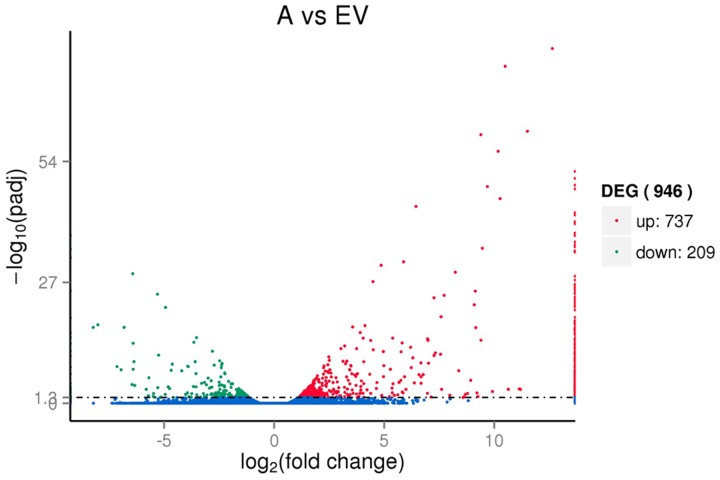
**Volcano plot of the DEGs between the transgenic *AANAT* and control switchgrass**.

To reveal the molecular events occurring during the transgenic process, the digital gene expression libraries were constructed using RNA from the pools of control and the transgenic RNA samples and sequenced using Illumina technology. After Illumina sequencing and the removal of adaptors and low-quality reads, approximately 76,976,922,81,777,632, and 84,557,876 reads were obtained for the three control replicates, and 76,912,922, 88,415,412, and 90,354,046 reads were obtain for the three replicates for the transgenic lines. We then mapped the clean reads to the transcriptome reference data, and a total of 54,349, 56,895 and 73,352 unigene sequences were identified for the control replicates, and 53,595, 56,080 and 57,158 unigene sequences were identified for the drought replicates. After calculating the expression level of each mapped unigene, a total of 946 unigenes were detected that had levels of expression that were significantly different between the transgenic and control libraries, including 737 up-regulated and 209 down-regulated unigenes (**Figure 3**). The significantly higher average FPKM of *AANAT* gene of transgenic lines (648.53) than that of control group (0.11).

The FPKM values were used to normalize gene expression levels and to compare their differences among transgenic lines and control. The percentage of high level expressed genes (FPKM beyond 15) in transgenic lines was slightly greater than in control samples, while that of low level expressed genes (FPKMs in the interval 0.1–3.57) in transgenic lines was relatively smaller than control samples (**Supplementary Table S5**).

### Functional Annotation and Classification of the DEGs by Gene Ontology Analysis

Among the differentially expressed unigenes, 558 genes were significantly up-regulated in the melatonin-rich switchgrass by more than twofold of the levels in the EV (*P* < 0.05). These genes included F-box protein (a gene for the controlled degradation of cellular protein); disease resistance protein (a resistance protein guard the plant against pathogens); abscisic stress-ripening protein 3 (an abscisic acid-, stress-, and ripening-induced protein); heat shock protein 83 (a gene for promoting the maturation), structural maintenance and proper regulation of specific target proteins involved for instance in cell cycle control and signal transduction.

**FIGURE 4:**
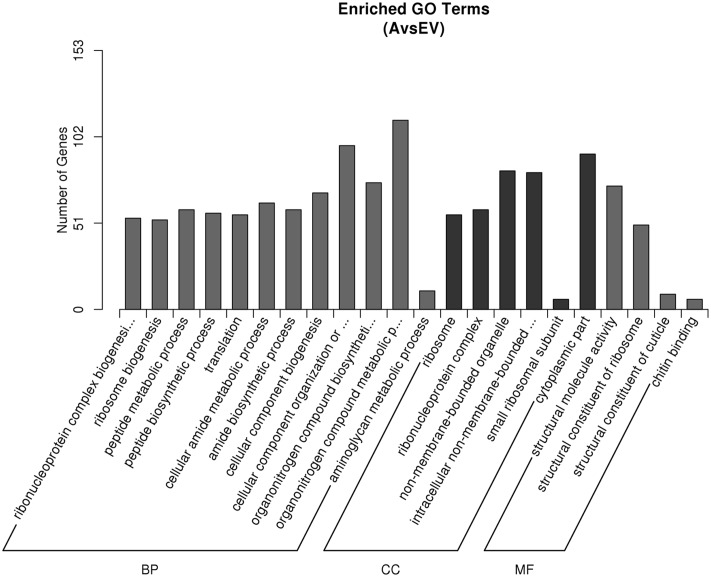
**Gene ontology (GO) classifications of DEGs across three comparisons.** The Y-axis represents the number of DEGs in a category. The results of transgenic A line vs. EV (control) are summarized in three main categories: biological process (BP), cellular component (CC), and molecular function (MF).

To identify the genes that are differentially expressed in transgenic lines, a functional categorization was carried out by GO analysis. A total of 511 unigenes, including 415 up-regulated genes and 96 down-regulated genes, were functionally assigned to the three categories of the GO database (**Figure 4**). The 2012 GO terms were functionally annotated with GO terms in transgenic and control groups. The GO terms of ‘organonitrogen compound biosynthetic process,’ ‘cellular amide metabolic process,’ ‘amide biosynthetic process,’ ‘aminoglycan metabolic process,’ and ‘peptide biosynthetic process’ in biological process were highly enriched in the DEGs (corrected *P*-value < 0.05), further suggesting the efficiency of the melatonin biosynthesis and the reliability of the gene expression data. Other terms, such as ‘cytoplasmic part,’ ‘non-membrane-bounded organelle,’ ‘ribonucleoprotein complex’ in cellular component, ‘structural molecule activity’ and ‘chitin binding’ in molecular function, were also significantly overrepresented (corrected *P*-value < 0.05). By comparing transgenic and control groups, 1687 up-regulated and 1094 down-regulated GO items.

### KEGG Pathway Analysis of the Melatonin-Related Genes

**FIGURE 5:**
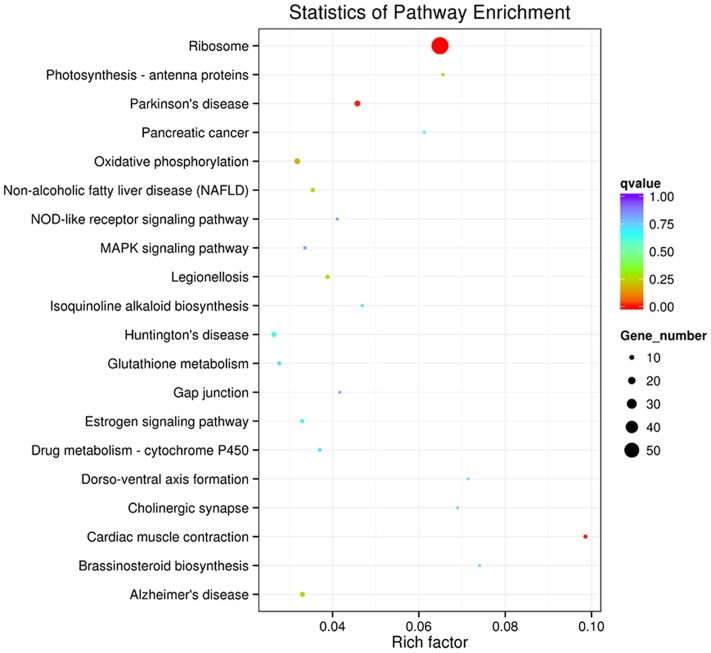
**Kyoto Encyclopedia of Genes and Genomes enrichments of the annotated DEGs across three comparisons.** The left Y-axis indicates the KEGG pathway. The X-axis indicates the Rich factor. A high q value is represented by blue, and a low q value is represented by red. (a) transgenic A line vs. EV (control); (b) transgenic H line vs. EV (control); (c) transgenic A vs. H line.

**FIGURE 6:**
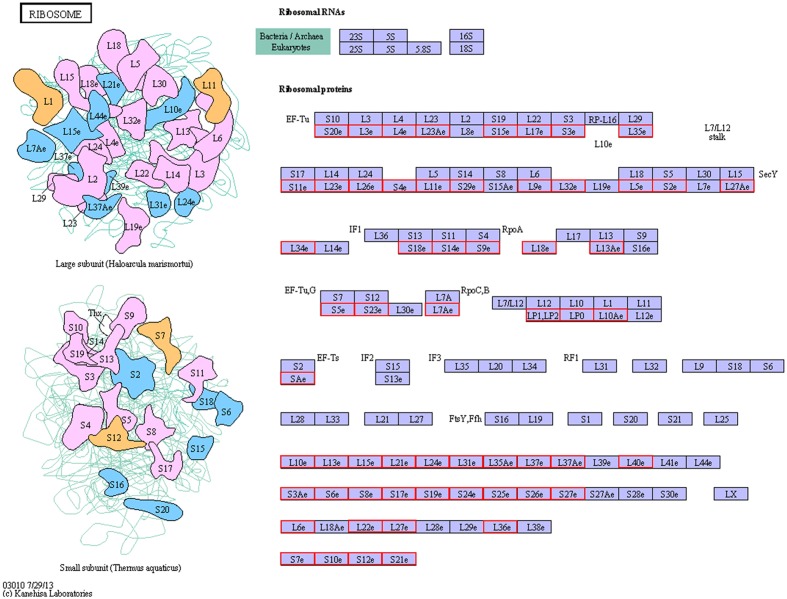
**The ribosome pathway enriched by KEGG analysis**.

To determine whether the melatonin-related genes engaged in specific pathways, the DEGs were used as objects to search against the KEGG pathway database. The top 20 obviously enriched pathways are shown in **Figure 5**. By comparing transgenic with control, ‘ribosome’ pathway enriched the most DEGs (**Figure 6**), followed by ‘oxidative phosphorylation,’ ‘Glutathione metabolism,’ ‘MAPK signaling pathway,’ ‘photosynthesis – antenna proteins’ and other pathways, suggesting that these pathways and processes might participate in the melatonin synthesis and metabolism (Supplementary file-KEGG).

### Transcription Factors

In this study, a total of 2214 transcription factors (TFs) were identified and classified into 79 different families, and the largest group of TFs was the MYB family (147, 6.64%), followed by NAC (108, 4.79%), Orphans (108, 4.88%), C_2_H_2_ (102, 4.62%), GRAS (101, 4.56%), C_3_H (95, 4.29%), bZIP (94, 4.29%), and WRKY (103, 6.71%; **Table 2**). These results further suggested that 33 DEGs encoding known or putative TFs were changed among transgenic and control groups, including the MYB, NAC, SNF2, and FAR1 TFs (**Table 2**).

**Table 2:** Selected genes about transcription factor with altered expression (*P* < 0.05) in the two groups.

Transcription factors	Genes	Description	FPKM	
			
			EV	A
AP2-EREBP	c52062_g7	Ethylene-responsive transcription factor RAP2-13-like	4.28	7.41
ARF	c53113_g1	Auxin response factor 16	14.39	22.89
ARR-B	c52945_g1	Two-component response regulator ARR10-like	1.79	3.08
C_2_H_2_	c52453_g3	C_2_H_2_-type zinc finger	11.57	28.20
CSD	c22802_g1	Cold shock domain-containing protein E1	0.00	0.32
CSD	c30734_g1	’Cold-shock’ DNA-binding domain	0.00	1.15
FAR1	c30195_g1	Protein FAR1-Related sequence 5 OS	0.71	0.01
G2-like	c50135_g1	GLK2 transcription factor	20.91	53.32
GRAS	c52194_g5	Heme binding	0.97	3.51
GRAS	c53142_g4	Heme binding	85.42	30.81
GRAS	c58702_g2	Heme binding	0.21	0.86
HMG	c33183_g2	HMG box-containing protein Drosophila melanogaster	0.00	0.40
Jumonji	c62341_g2	Putative transcription factor 5qNCA, contains JmjC domain	4.48	0.41
LIM	c52258_g1	LIM domain-containing protein WLIM1 OS	20.25	24.29
LOB	c57227_g1	LOB domain-containing protein 37-like	62.87	162.85
MYB	c55204_g2	MYB44-like	49.42	18.80
MYB	c60941_g1	MYB superfamily, myb proto-oncogene protein	101.44	26.08
MYB	c63952_g2	MYB DNA-binding domain superfamily protein	0.03	0.33
MYB	c50835_g3	MYB proto-oncogene protein	183.08	213.97
MYB	c61614_g1	MYB proto-oncogene protein	137.17	153.77
MYB	c64075_g2	MYB proto-oncogene protein	6.36	10.69
NAC	c58322_g4	NAC domain transcription factor superfamily protein	68.51	189.30
NAC	c46269_g1	NAC domain-containing protein	5.72	1.39
Orphans	c53871_g2	Zinc finger protein CONSTANS-LIKE 3-like	180.04	388.03
Orphans	c57774_g5	Zinc finger protein CONSTANS-LIKE 16-like	94.83	212.30
Orphans	c61529_g5	B-box zinc finger protein 25-like	109.68	502.47
Orphans	c87095_g1	B-box zinc finger	0.00	2.01
SNF2	c50837_g2	SWI/SNF-related matrix-associated actin-dependent regulator of chromatin subfamily A member 3-like 3	1.27	0.13
SNF2	c42819_g1	SNF2 domain-containing protein	0.13	0.78
SWI/SNF-BAF60b	c38813_g1	SWIB domain-containing protein 1	0.00	0.83
TAZ	c57461_g1	BTB/POZ and TAZ domain-containing protein 1	6.89	23.44
TRAF	c5721_g1	BTB/POZ domain//Zinc finger, C_2_H_2_ type	0.00	0.45
WRKY	c56069_g1	WRKY transcription factor 53 isoform X2	6.72	1.77


### Validation of Gene Expression Profiles Using RT-qPCR

The 12 DEGs were randomly selected for qRT-PCR. Histograms were generated by comparing the FPKM determined by transcriptome analysis and qRT-PCR. A highly significant correlation (*R*^2^ = 0.703, *P* < 0.01) was found between the qPCR and RNA-Seq, indicating reproducibility and credible RNA-seq data (**Supplementary Figure S3**).

## Discussion

Although, a growing body of molecular and gene expression research regarding melatonin has been documented on model plants (Kang et al., 2010; Park et al., 2012; Byeon et al., 2013b; Weeda et al., 2014), the expression profiling of genes on melatonin biosynthesis and metabolism in forage grass has not yet been investigated, which was potentially valuable for the molecular breeding of the switchgrass as an superior bioethanol grass. In this study, to offer an initial insight into the related genes of melatonin in plants, we conducted a RNA-seq analysis to generate the global transcriptomic profile in transgenic switchgrass plants overexpressing the sheep *AANAT* gene.

Overexpression of the o*AANAT* gene, encoding the key enzyme during melatonin biosynthesis process, significantly enhanced melatonin contents (over threefold of the EV from our previous work, *P* < 0.01) and promoted growth (shoot height, root length, stem diameter, leaf size) and reproductive (spike length) processes in transgenic switchgrass (**Table 1**). Through the classic view supports the last HIOMT/ASMT enzyme is rate-limiting during the biosynthesis of melatonin, the promotional roles of AANAT/SNAT were also reported in other species, e.g., the *oAANAT* transgenic ‘micro-tom’ tomato have higher melatonin levels than control (Wang et al., 2014). The positive regulatory role of melatonin on growth and development was confirmed in other transgenic melatonin-rich plants (Byeon and Back, 2014; Wang et al., 2014). For example, the transgenic *oAANAT* rice exhibited altered height, biomass and panicle numbers per plant, suggesting that melatonin took part in plant growth and reproduction (Byeon and Back, 2014). Moreover, exogenous melatonin treatments also enhanced root regeneration, photosynthetic pigments, total carbohydrates and biomass in the *Chenopodium rubrum* (Kolár et al., 2003), cherry rootstock (Sarropoulou et al., 2012); cucumber (Zhang et al., 2013), and soybean (Wei et al., 2015). The low concentrations melatonin increases photosynthetic activity by inducing porphyrin and chlorophyll biosynthesis, in contrast, high concentrations of melatonin induces the synthesis of proline and carbohydrate, which are beneficial for the osmoregulation of plants under stresses (Sarropoulou et al., 2012). Moreover, the stimulation of root generation and vitality and addition of the root/shoot ratio under melatonin treatment supported its effect on strengthening cucumber roots (Zhang et al., 2013). Furthermore, we detected the earlier flowering of transgenic *oAANAT* switchgrass than EV. Several flower-specific genes, *FLC* (Flowering Locus C, c52347_g1), *AP2* (APETALA2, c34036_g2, c48942_g1), *DELLA* (c47591_g2), differentially expressed between transgenic *oAANAT* and EV plants (*P* < 0.05). However, more flowering related genes did not showed significantly regulated by overexpression of *oAANAT* genes (*LFY*, *CO*, *TFL*, *AG*, *AP3*). This might be attributed by the discrepancies of the gene expressions between plant stems and inflorescences, considering that the large number of putative floral-specific transcripts were detected in flowers of sweet potato using RNA-seq (Tao et al., 2013). The striking promotions on development patterns of stems, leaves and roots and alteration of flowering suggested the possible involvement of the melatonin in these physiological actions (Park, 2011; Arnao and Hernández-Ruiz, 2014; Hardeland, 2016).

Transcriptomic analysis exhibited that the large number of 946 DEGs in the melatonin-rich switchgrass comparing to the control (**Figure 3**). Among the DEGs, functional patterns of melatonin were divided into two main aspects identifying by gene ontology analysis: the growth regulator and stress tolerance. The growth related function of melatonin was supported by the GO terms of plant development (‘post-embryonic root development,’ ‘seed germination,’ ‘seedling development,’ and ‘pollen tube development’) and biosynthetic processes (‘organonitrogen compound biosynthetic process,’ ‘amide biosynthetic process,’ and ‘peptide biosynthetic process’). The genes encoding for signaling regulation and TFs such as MYB domain-containing protein, NAC and C_2_H_2_ type protein, leucine-rich repeat and zinc-finger genes were involved in plant growth and metabolisms (**Table 2**). These differentially expressed TFs were also detected in the other transcriptome analysis of growth related phenotypes in switchgrass (Palmer et al., 2012; Wang et al., 2012). The DREB subfamily, AP2 TFs, several different classes of zinc finger TFs (C_2_H_2_), auxin responsive TFs (ARF), Myb family TFs, and NAC were identified in the *Arabidopsis* dormancy related gene set (Wang et al., 2012). The APETALA2/ethylene response factor (AP2/ERF) superfamily of TFs plays essential roles in the regulation of various growth and developmental programs including stress responses (Wuddineh et al., 2015). This may be closely associated with the gene-regulation capacity of melatonin as a plant growth regulator (Park, 2011; Arnao and Hernández-Ruiz, 2014).

Furthermore, the stress tolerance function of melatonin was evidenced by the GO terms of ‘response to oxidative stress’ and ‘cellular response to stress.’ The F-box protein (the controlled degradation of cellular protein); disease resistance protein (a resistance protein guard the plant against pathogens); abscisic stress-ripening protein 3 (an abscisic acid-, stress-, and ripening-induced protein); heat shock protein 83 (a gene for promoting the maturation) involving for cell cycle control and signal transduction were differentially expressed in transgenic *oAANAT* switchgrass (**Table 2**). Similarly, the oxidative resistances were also detected in transgenic rice (Kang et al., 2010) and agrochemicals (Park et al., 2012) owing to the increased levels of endogenous melatonin. The promotion of the maturation, structural maintenance and proper regulation of specific target proteins was consistent with the result of a previous study in which a microarray analysis on the *AANAT* overexpressed rice, suggesting that melatonin is involved with stress responses (Byeon et al., 2013b). In addition, exogenous melatonin enhances abiotic tolerance (water, cold, salt stresses) in various plants (Zhang et al., 2013; Fan et al., 2015; Wei et al., 2015). The inhibitory effects of salt stress on gene expressions related to binding, oxidoreductase activity/process, and secondary metabolic processes under salt stress were alleviated by melatonin in soybean (Wei et al., 2015). Melatonin achieves its promotional roles by influencing gene expressions involved in metabolic activities: nitrogen metabolism, major carbohydrate metabolism, hormone metabolism, and secondary metabolism (Paredes et al., 2008; Nawaz et al., 2015).

Kyoto Encyclopedia of Genes and Genomes maps provided much information on exploration of metabolic pathways involved in melatonin and deconstruction of its biological functions. Many differentially expressed unigenes were enriched into the biosynthetic pathways of secondary metabolite (phenylpropanoids, flavonoids, steroids, stilbenoid, diarylheptanoid, and gingerol). Plant steroid is used as signaling molecules for physiological and developmental regulation and offers exciting potentials for enhancing crop yield (Vriet et al., 2012). In addition, signaling pathways (MAPK signaling pathway, estrogen signaling pathway) were significantly unregulated as well (**Figure 5**). Taking the MAPK (Mitogen-activated protein kinase) pathway as example, responses to various biotic and abiotic stresses as an integral component of cellular signaling during mitogenesis and differentiation of mitotic cells (Atkins et al., 1998; Group et al., 2002; Supplementary file-KEGG). The decades of secondary metabolic process and signal pathways related genes were differentially expressed in both endogenous melatonin-rich transgenic rice (Byeon et al., 2013b) and exogenous melatonin treatment (Zhang et al., 2014). These significantly altered expressions of metabolic pathways were not completely consistent between endogenous and exogenous melatonin treatment: the method of spraying leaves or coating seeds with melatonin merely drives short-term changes in morphological and physiological traits, while overexpression of endogenous melatonin gene enables sustainable synthesis of melatonin during the lifespan (Park, 2011). Besides, overexpression genes of the melatonin biosynthesis key enzyme participates in the *de novo* synthesis and is possibly involved in much more metabolism pathways than those from exogenous treatment, thus providing valuable information for the elucidation the biosynthesis pathway and functional mechanism of melatonin in plants (Kang et al., 2011, 2013; Byeon et al., 2013a,b; Zhang et al., 2016).

Previous studies have revealed that melatonin had significant effect in regulating hormones (such as ABA, GA_4_) in plant response to salinity and drought stress (Li et al., 2012; Zhang et al., 2016). Melatonin shared the common substrate (tryptophan) with IAA, however, the relationship of biosynthesis between melatonin and IAA is still in suspense and the independent relationship has been proposed in *Arabidopsis* root system architecture (Pelagio-Flores et al., 2012; Arnao and Hernández-Ruiz, 2014; Wei et al., 2016). In our study, the auxin-responsive protein IAA genes differentially expressed in the melatonin-rich switchgrass comparing with the control, and possibly resulted from the competitive relation for the same biosynthesis substrate. Thus, genome-wide expression analysis of melatonin-related genes in response to plant hormones supported the crosstalk between melatonin and IAA biosynthesis processes, and the intern mechanism of the relative expression needs more clues from other direct analysis of hormones treatments (Zhang et al., 2014; Wei et al., 2016).

## Conclusion

This study is the first report of the transcriptomic profile of endogenous melatonin effects on in bioenergy crop switchgrass using RNA-seq technology. Switchgrass is an identified model species for bioethanol but the genetic background and genome information are not well-established, and these transcriptomic datasets will provide fundamental information and serve as new tools to genetically dissect melatonin-mediated pathways in other common grasses. The analysis based on DEGs reveals broad roles of melatonin in regulating plant growth, development and defense systems. Furthermore, the expression of many genes involved in signaling regulation such as MAPK signaling pathway, was also altered in response to the transgenic switchgrass of overexpression of melatonin biosynthesis gene. Taken together, studies on sequencing of transgenic melatonin-rich switchgrass suggests that melatonin plays a critical role in promotion of plant growth and may facilitate identification of melatonin’s functions in plants.

## Author Contributions

Conceived and designed the experiments: YZ, FY. Performed the experiments: SY, YH, CG, SL, DT. Analyzed the data: SY, CG, XC. Wrote the paper: SY, YH, YZ. All authors reviewed and approved the final manuscript.

## Conflict of Interest Statement

The authors declare that the research was conducted in the absence of any commercial or financial relationships that could be construed as a potential conflict of interest.

The reviewer Y-DG declared a shared affiliation, though no other collaboration, with the authors to the handling Editor, who ensured that the process nevertheless met the standards of a fair and objective review.

## Footnotes

1 http://www.ncbi.nlm.nih.gov/bioproject

2 http://www.genome.jp/kegg/
