# Supplementary material for: RNA-seq Analysis of Overexpressing Ovine AANAT Gene of Melatonin Biosynthesis in Switchgrass
Source: Front Plant Sci. 2016 Aug 31;7:1289. doi: 10.3389/fpls.2016.01289 (PMC5026198; doi:10.3389/fpls.2016.01289)

Supplementary Tables and Figures

RNA-seq analysis of overexpressing ovine *AANAT* gene of melatonin biosynthesis in switchgrass

Shan Yuan[1], Yanhua Huang[1,2], Cong Guan[1], Sijia Liu[1], Xin Cui[1], Danyang Tian[1], Yunwei Zhang[1,3,4]*, Fuyu Yang[1,5]*

1 College of Animal Science and Technology, China Agricultural University, Beijing, China; 2 College of Agriculture, China Agricultural University, Beijing, China; 3 Beijing Key Laboratory for Grassland Science, China Agricultural University, Beijing, China; 4 National Energy R&D Center for Biomass (NECB); 5 Beijing Sure Academy of Biosciences

**Correspondence and requests for materials should be addressed to Y. Z (Email: zywei@126.com Tel: +86-10-62733572 Fax: +86-10-62733572). F. Y (Email: Yun Tel: +86-10-010-62733052 Fax: +86-10-62734252).*

Supplementary Table S1 Oligonucleotide primers used in RT-PCR analysis

No.	Unigene	Forward primer (5'-3')	Reverse primer (5'-3')
P8	c65591_g1	GACCCAGTTGACCACTCTTTAC	GCAGCCTCAACCAAGTAAGT
P10	c65662_g1	CCCGAACCACGATGAACTATG	GCATCTTCCGCATCAATCTCTA
P14	c56332_g2	CCCATCTTTCCCAGATCACAA	TCTGGCGAGATAGAGGTCAT
P17	c49639_g1	ATCAACCAGCTCGGGTACTA	AGCATGCACATCCATCCATAC
P19	c63279_g3	GCGATCCAGGGAGAGTTATTC	GCGATCCAGGGAGAGTTATTC
P20	c50061_g4	ACGGTTCACTCCCTAGTCTC	GGGATCGTGATGTCCAGAATG
P21	c48823_g1	GCCATGTTTCAGGTGACTCT	CATGTCTGGATGAACTGGAAGG
P26	c53254_g2	CTGGAGCTTCTCCATGTTGAT	ACACAAGAGAGCCAGAGAGA
P29	c49628_g1	GACTGAGTGGCACATAGGAAA	CGAGACACAGATGGTGTAGAAC
P30	c102099_g1	TGCCATCGTGGTAGTGTTTG	GCTCCTGACACCAAAGGATAAG
P34	c27561_g1	GAGAGCCGCAACAAGAGAAA	CTGCTTGTCGGTATGAGGTATG
P36	c55261_g4	TCGGTGAGTTTGGAGGTTTG	AGCACCCTACTGTTTGTCTTC

Supplementary Table S2 Summary of Illumina transcriptome sequencing. EV: expressing the empty vector only, A: transgenic *oAANAT* line.

Sample	Raw Reads	Clean Reads	Clean Bases	Error(%)	Q20(%)	Q30(%)	GC Content(%)
A_1	80430918	76912922	11.54G	0.01	97.57	94.08	59.66
A_2	94162412	90354046	13.55G	0.01	97.52	93.91	59.57
A_3	92073472	88415412	13.26G	0.01	97.68	94.29	59.69
EV_1	80891154	76976922	11.55G	0.01	97.24	93.14	58.71
EV_2	87541130	81777632	12.27G	0.01	97.22	93.26	57.87
EV_3	89891632	84557876	12.68G	0.01	97.18	93.18	55.11

Supplementary Table S3 Summary of the switchgrass transcriptome.

Category	Number				Total	Mean length	N50	Total nucleotides
	200-500	500-1k	1k-2kb	>2kbp				
Transcripts	111059	57802	55055	40953	264869	1052	1792	278653546

Unigenes	84604	25347	14922	10811	135684	716	1243	97207303

Supplementary Table S4 Summary of the functional annotation of assembled unigenes.

Public database	Number of unigenes	Percentage (%)
Annotated in NR	56968	41.98
Annotated in NT	60512	44.59
Annotated in KO	19493	14.36
Annotated in SwissProt	38266	28.2
Annotated in PFAM	39624	29.2
Annotated in GO	41494	30.58
Annotated in KOG	19077	14.05
Annotated in all Databases	8900	6.55
Annotated in at least one Database	77421	57.05
Total Unigenes	135684	100

Supplementary Table S5 Statistics of genes in different expression-level interval. EV: expressing the empty vector only, A: transgenic *oAANAT* line.

FPKM	EV	A
0-0.1	69928(51.54%)	71734(52.87%)
0.1-0.3	10145(7.48%)	9312(6.86%)
0.3-3.57	29150(21.48%)	28221(20.8%)
3.57-15	13661(10.07%)	13380(9.86%)
15-60	9312(6.86%)	9574(7.06%)
>60	3488(2.57%)	3463(2.55%)

Supplementary Fig. S1 Summary of the functional annotation of assembled unigenes among different databases.

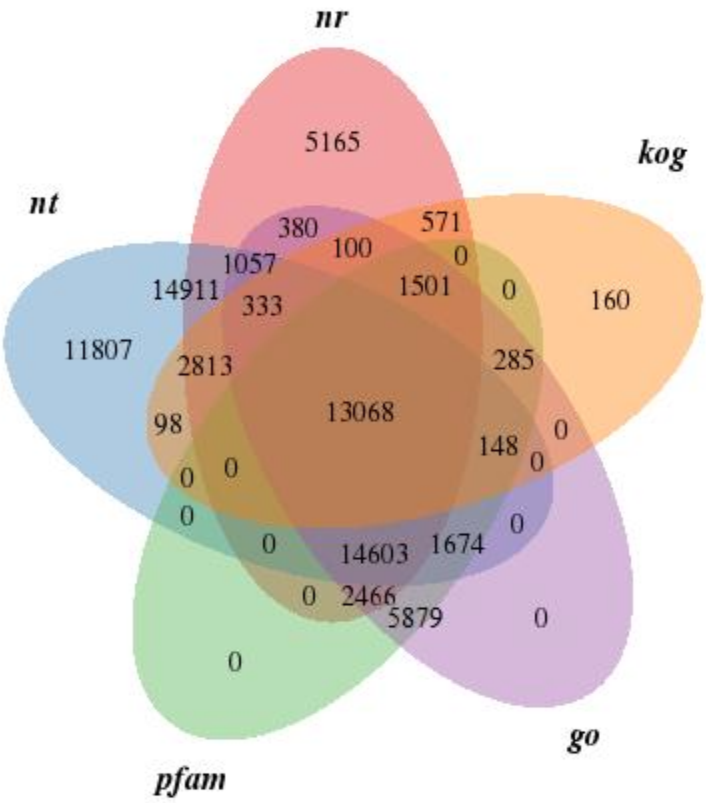

Supplementary Fig. S2 Functional classification of the assembled unigenes. (a) Functional classification of the assembled unigenes based on Gene Ontology (GO) categorization. The results are summarized in three main GO categories: biological processes, cellular components and molecular functions. The x-axis indicates the subcategories, and the y-axis indicates the numbers related to the total number of GO terms present; the unigene numbers that are assigned the same GO terms are indicated at the top of the bars. (b) Histogram of eukaryotic orthologous groups (KOG) classification. The unigenes were aligned to the KOG database to predict and classify possible functions.

(a)

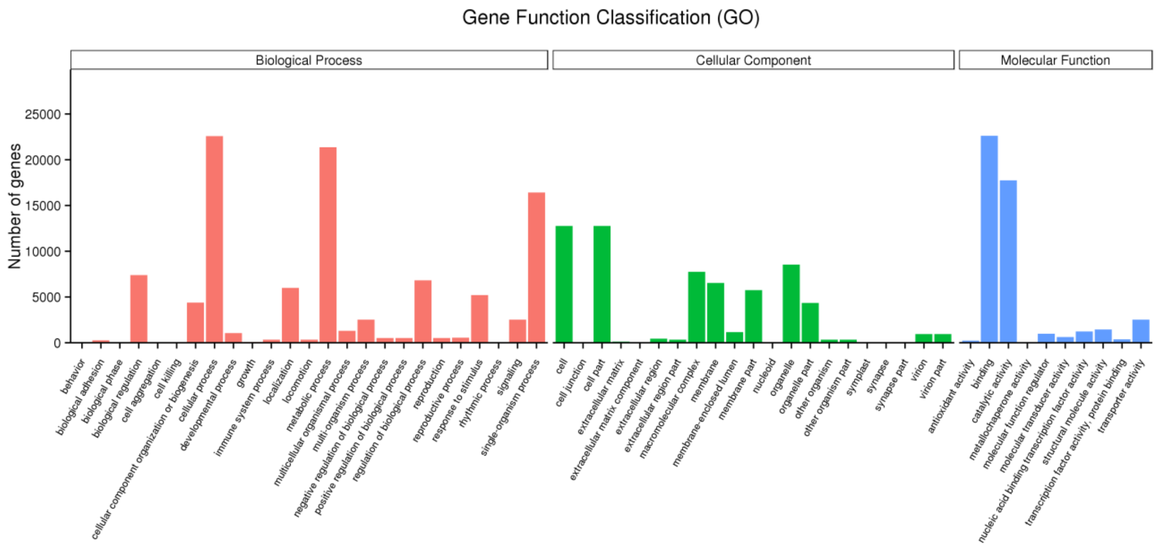
Gene Function Classification (GO)
Biological Process
Cellular Component
Molecular Function
Number of genes
25000
20000
15000
10000
5000
0
behavior
biological adhesion
biological phase
biological regulation
cell aggregation
cell killing
cellular component organization or biogenesis
cellular process
developmental process
growth
immune system process
localization
locomotion
metabolic process
multicellular organismal process
multi-organism process
negative regulation of biological process
positive regulation of biological process
regulation of biological process
reproduction
reproductive process
response to stimulus
rhythmic process
signaling
single-organism process
cell
cell junction
cell part
extracellular matrix
extracellular matrix component
extracellular region
extracellular region part
macromolecular complex
membrane
membrane-enclosed lumen
membrane part
nucleoid
organelle
organelle part
other organism
other organism part
symplast
synapse
synapse part
virion
virion part
antioxidant activity
binding
catalytic activity
metallochaperone activity
molecular function regulator
molecular transducer activity
nucleic acid binding transcription factor activity
structural molecule activity
transcription factor activity, protein binding
transporter activity

(b)

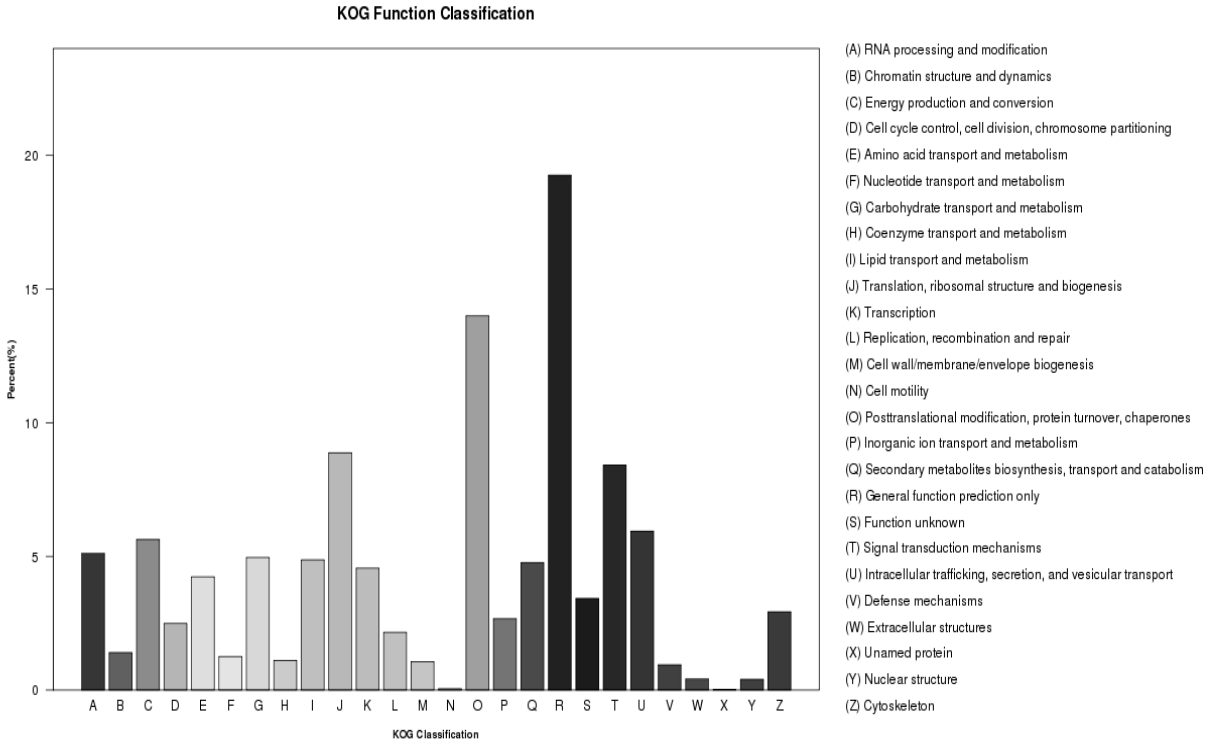
KOG Function Classification
Percent(%)
20
15
10
5
0
A B C D E F G H I J K L M N O P Q R S T U V W X Y Z
KOG Classification
(A) RNA processing and modification
(B) Chromatin structure and dynamics
(C) Energy production and conversion
(D) Cell cycle control, cell division, chromosome partitioning
(E) Amino acid transport and metabolism
(F) Nucleotide transport and metabolism
(G) Carbohydrate transport and metabolism
(H) Coenzyme transport and metabolism
(I) Lipid transport and metabolism
(J) Translation, ribosomal structure and biogenesis
(K) Transcription
(L) Replication, recombination and repair
(M) Cell wall/membrane/envelope biogenesis
(N) Cell motility
(O) Posttranslational modification, protein turnover, chaperones
(P) Inorganic ion transport and metabolism
(Q) Secondary metabolites biosynthesis, transport and catabolism
(R) General function prediction only
(S) Function unknown
(T) Signal transduction mechanisms
(U) Intracellular trafficking, secretion, and vesicular transport
(V) Defense mechanisms
(W) Extracellular structures
(X) Unamed protein
(Y) Nuclear structure
(Z) Cytoskeleton

Supplementary Fig. S3 Validation of RNA-Seq analysis by reverse transcription-quantitative polymerase chain reaction (RT-qPCR). FPKM (fragments per kilobase of exon per million fragments mapped) values obtained with RNA-Seq and qPCR values in the analysis of selected genes in the three assayed groups: EV: expressing the empty vector only, A: transgenic *oAANAT* line. Error bars represent the standard error for three independent experimental replicates.

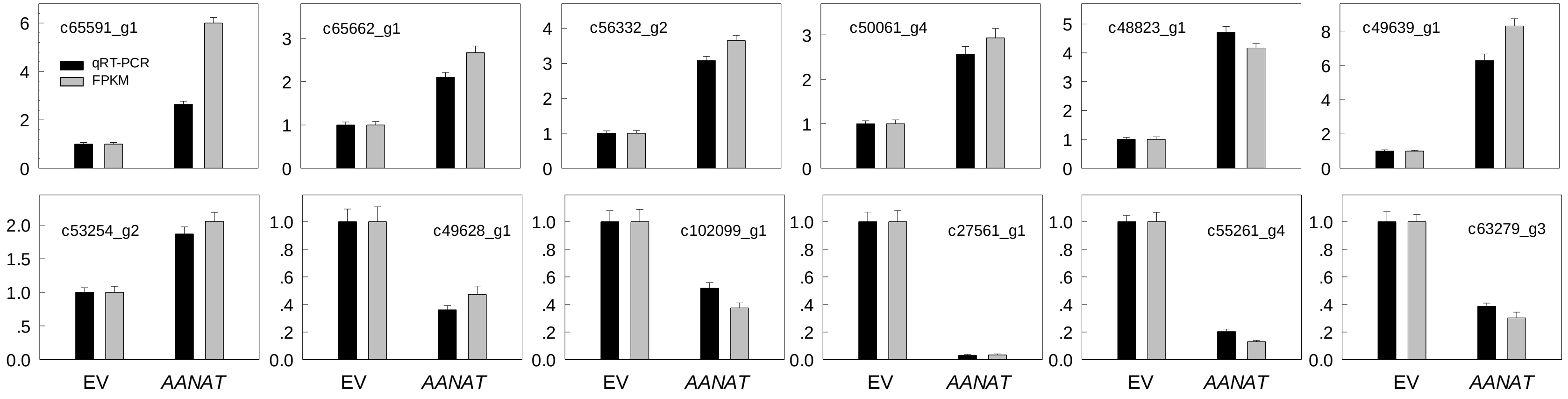

Supplement: Supplementary file 1 [file Table_1.PDF]
